# Control of Body Temperature during Physical Exercise

**DOI:** 10.5935/abc.20190081

**Published:** 2019-05

**Authors:** Ricardo Luiz Damatto,, Marcelo Diarcadia Mariano Cezar, Priscila Portugal dos Santos

**Affiliations:** 1 Sociedade Cultural e Educacional de Itapeva - Educação Física, Itapeva, SP - Brazil; 2 Universidade Estadual Paulista Júlio de Mesquita Filho Campus de Botucatu - Faculdade de Medicina, Botucatu, SP - Brazil

**Keywords:** Exercise, Rats, Rats, Inbred SHR/physiology, Body Temperature Regulation

Physical exercise is currently recommended for health promotion and as a non-pharmacological treatment of cardiovascular diseases. Regular exercise results in improved body composition and physical capacity, as well as decreased insulin resistance and arterial hypertension, leading to ameliorated quality of life.^1^

During exercise, heat is a by-product of metabolism, which leads to increased body temperature. However, the human body needs to maintain a stable temperature, around 37ºC, using neural and cardiovascular mechanisms. The temperature-regulating center is found in the anterior hypothalamus. It receives information on the ambient temperature, through the skin thermoreceptors, and on the internal temperature, through the hypothalamic thermoreceptors. Thus, the hypothalamus promotes appropriate responses of heat generation or dissipation, which involve arteriovenous redistribution of blood.^2^ Therefore, individuals with cardiovascular comorbidities such as type II diabetes, hypercholesterolemia and arterial hypertension may present impairment of thermoregulation mechanisms.^3^

In order to study and evaluate hypertension, Spontaneously Hypertensive Rats (SHR) are commonly used as a model, since they resemble the condition found in humans.^4,5^ Therefore, the Gomes et al.^6^ used SHR rats to evaluate the effects of low-intensity physical exercise training on thermal balance.

After 12 weeks of exercise protocol, the Gomes et al.^6^ showed a reduction in blood pressure in trained SHR. In addition, trained SHR presented lower skin temperature than trained Wistar. This show an impaired heat dissipation in SHR. However, physical exercise did not influence the promotion of positive adaptations on thermoregulation.^6^

In humans, heat dissipation responses involve increased sweating, as the main mechanism, and cutaneous active vasodilation.^2^ Thermoregulatory responses in rats are different. Cutaneous vasodilation of the tail is the main mechanism of heat dissipation in this species, accounting for 40% of heat loss during exercise.^7^ This mechanism can be activated by central cholinergic stimulation via modulation of arterial baroreceptors by increasing the blood flow of the rat´s tail.^8,9^ Additionally, vasodilation of the skin of the feet, the evaporation of saliva spread onto the body surface, the evaporation of water from the respiratory tract and even voluntary urination associated with urine spreading activity may also contribute to the total heat dissipation.^10^

Considering the relationship between the cardiovascular system and the regulation of body temperature, hypertension can affect the mechanisms of heat dissipation. In SHR rats, for example, decreased baroreceptor sensitivity, sympathetic hyperactivity, which leads to increased peripheral resistance, and endothelial dysfunction may impair cutaneous vasodilation of the tail and, consequently, heat dissipation.^7,9^

In fact, the Gomes et al.^6^ found lower skin temperature in trained SHR than in trained Wistar. This shows a lower heat dissipation in hypertensive animals during exercise. However, the author did not observe alterations in the internal temperature, heat dissipation threshold, sensitivity and cumulative heat normalized by the work. One possible explanation is that other mechanisms of heat dissipation, besides cutaneous vasodilation of the tail, may have been used by these animals.
